# P-2013. Improving Access to *COVID-19* Treatment for Longterm Care (LTC) Residents: An Integrated Operational Approach

**DOI:** 10.1093/ofid/ofae631.2170

**Published:** 2025-01-29

**Authors:** Ajit Johal, Mark Zhou

**Affiliations:** Immunize.io Health Association , Vancouver, British Columbia, Canada; Immunize.io Health Association, Vancouver, British Columbia, Canada

## Abstract

**Background:**

Residents residing in Long-term care (LTC) facilities suffer disproportionately from severe outcomes of COVID-19. Antiviral treatment remains underutilized despite ongoing evidence in reducing severe disease in this high-risk group. In this quality improvement study conducted in British Columbia, Canada, barriers to treatment access were evaluated and addressed with educational resources to support the implementation of protocols within the LTC setting.

Concern regarding COVID-19 outbreaks in Longterm Care

**Figure:**
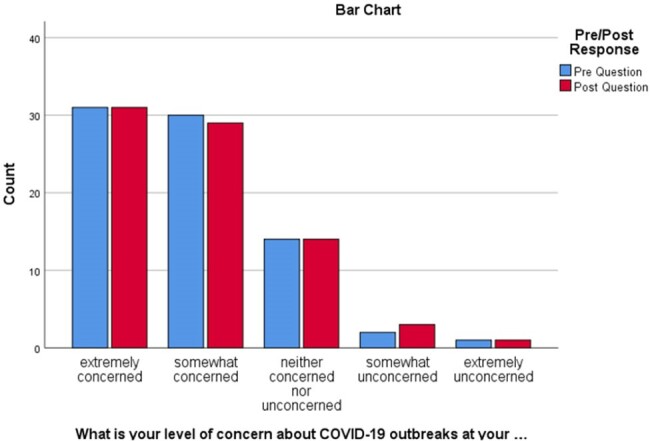

Bar Chart categorizing pre- and post-responses from LTC staff regarding the concern of COVID-19 outbreaks at their facility during the study period.

**Methods:**

Educational resources were developed by the study team and disseminated to Long-term care staff (n=564). Specifically, information for patients and their families about COVID-19 antiviral treatment, care plan templates for residents who opt in for treatment, and digital education videos for staff to support implementation of the aforementioned resources within the LTC setting. During the study period, January to April 2024, LTC staff were surveyed to determine the the usefulness of the resources.

Comfort level managing drug-drug interactions

**Figure:**
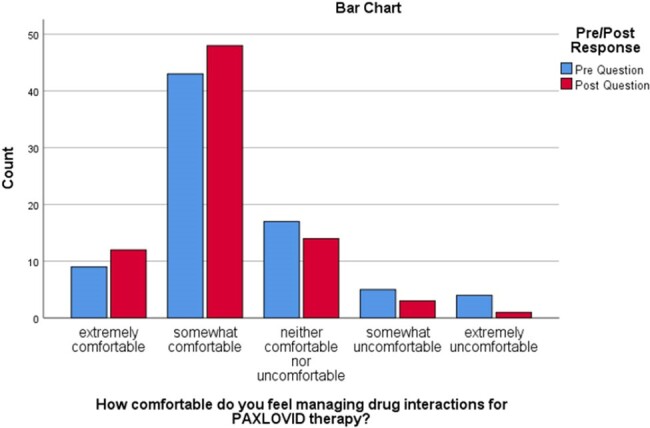

Bar Chart categorizing pre- and post-responses reflecting LTC staff comfort levels in managing drug interaction with PAXLOVID therapy before and after the intervention.

**Results:**

LTC staff respondents (n=78) consisted of Nurses (n=54), Pharmacists (n=13), and other staff (n=11) across LTC facilities (n=43) in Vancouver, BC. Most respondents (n= 31) expressed extreme concern for COVID-19 outbreaks in their facility, which remained unchanged during the study period (p=0.848). They reported unclear patient eligibility and drug-drug interactions as the biggest barriers to accessing COVID-19 treatment for their residents. After reviewing all educational materials with their respective facilities, respondents showed statistically significant changes in their comfort levels in discussing COVID-19 treatment with residents and their families (p< 0.01). However, no statistically significant change was seen in the pre-and post-evaluation in comfort levels managing drug interactions with COVID-19 antiviral therapy (p=0.155).

Comfort level discussing PAXLOVID treatment

**Figure:**
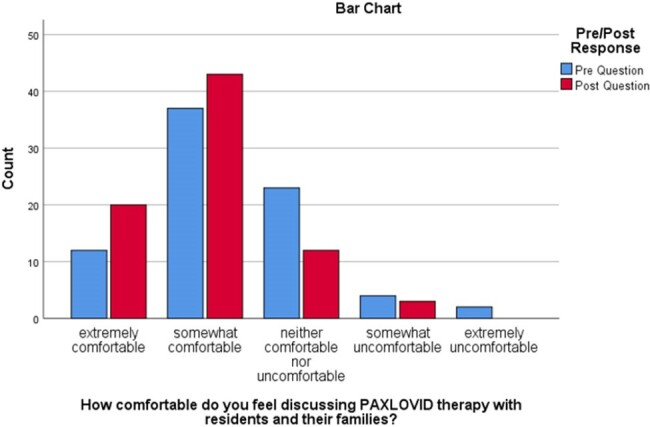

Bar Chart categorizing pre- and post-responses reflecting LTC staff comfort levels in discussing PAXLOVID therapy with residents and their families

**Conclusion:**

COVID-19 remains a concern in LTC settings, with staff encountering barriers to accessing antiviral treatment for their residents. An integrated operational approach, defined as curated resources highlighting information for residents and their families and care plan templates to support implementation, may help support timely access to antiviral treatments in this setting.

Perceived Barriers to COVID-19 treatment in LTC

**Figure:**
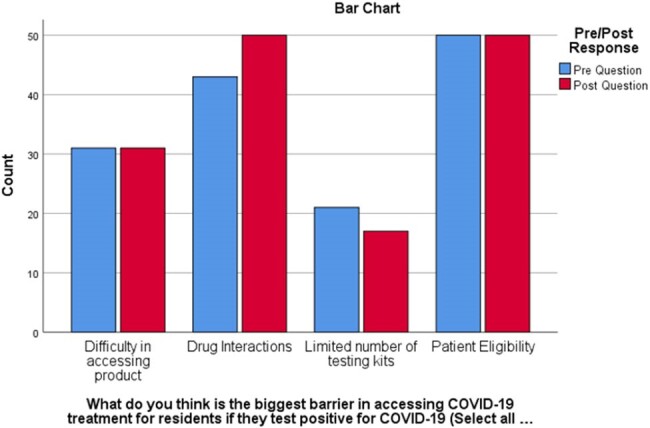

Bar Chart categorizing pre- and post-responses reflecting LTC staff perceived barriers in accessing COVID-19 treatment, in the event of a confirmed positive test

**Disclosures:**

Ajit Johal, BSP BCPP RPh, GSK: Grant/Research Support|GSK: Honoraria|Merck: Grant/Research Support|Merck: Honoraria|Moderna: Honoraria|Pfizer: Grant/Research Support|Pfizer: Honoraria|Sanofi Pasteur: Honoraria|Seqirus: Honoraria|Valneva: Honoraria

